# Supplementary material for: Concerted roles of LRRTM1 and SynCAM 1 in organizing prefrontal cortex synapses and cognitive functions
Source: Nat Commun. 2023 Jan 28;14:459. doi: 10.1038/s41467-023-36042-w (PMC9884278; doi:10.1038/s41467-023-36042-w)
Supplement: Supplementary file 1 — Supplementary Information [file 41467_2023_36042_MOESM1_ESM.pdf]

Supplementary Information

Concerted roles of LRRTM1 and SynCAM 1 in organizing prefrontal cortex synapses and cognitive functions

Karen Perez de Arce, Adema Ribic, Dhrubajyoti Chowdhury, Katherine Watters,
Garth J. Thompson, Basavaraju G Sanganahalli, Elizabeth T. C. Lippard, Astrid Rohlmann,
Stephen M. Strittmatter, Markus Missler, Fahmeed Hyder, and Thomas Biederer

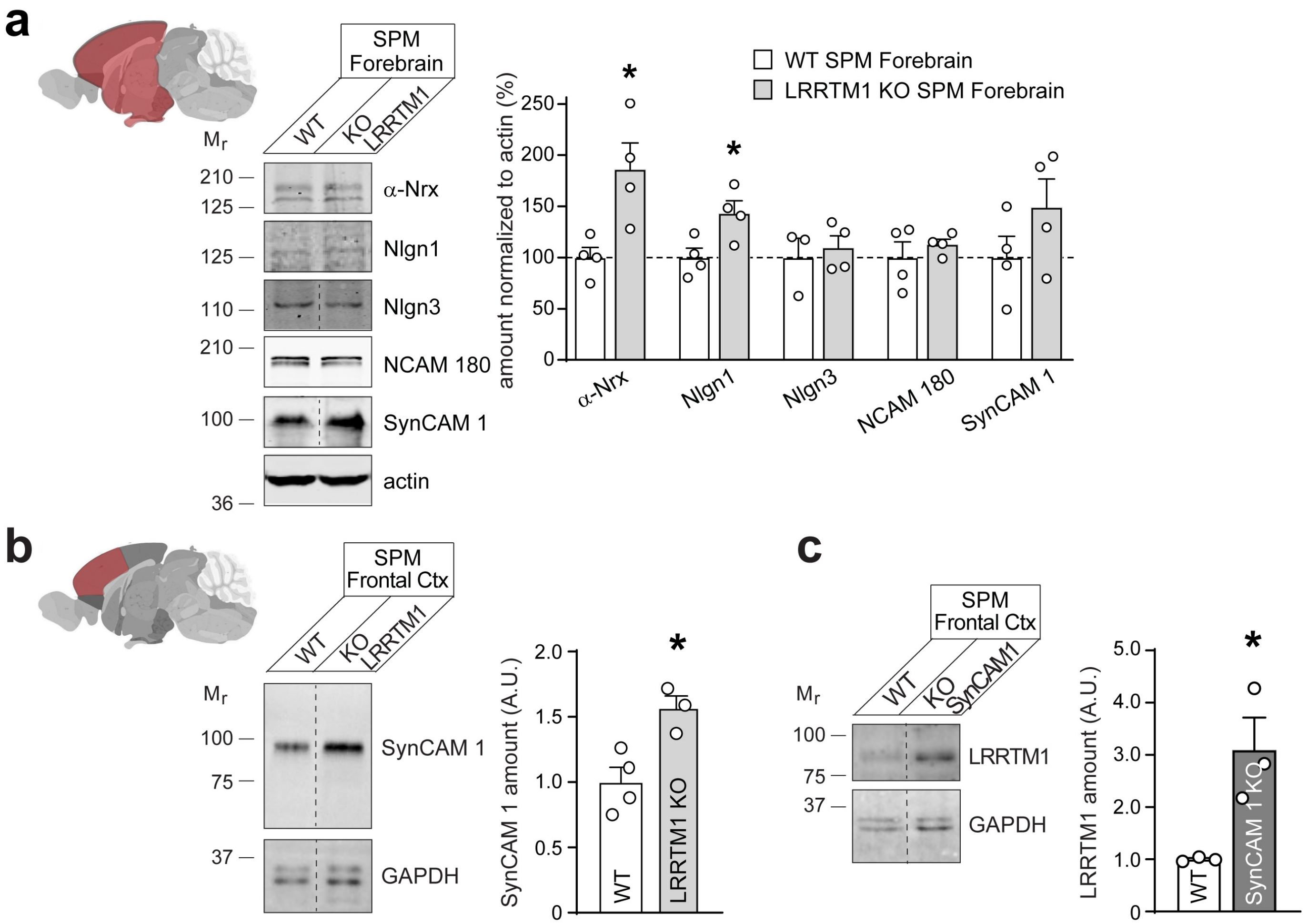

Supplementary Figure 1. Expression of synapse organizers in forebrain and frontal cortex synaptic plasma membrane preparations upon LRRTM1 loss.

a Increase in α-Neurexins (α-Nrx1) and Neuroligin 1 (Nlgn1) in synaptic plasma membranes (SPM) prepared from the total forebrain of LRRTM1 KO mice compared to WT littermates. Left, diagram with the forebrain area dissected for fractionation marked in red. Center, immunoblots of WT and LRRTM1 KO forebrain SPM probed for the indicated proteins. 15 μg protein was loaded per lane. Dashed lines separate lanes from the same blot exposure. Right, quantification of immunoblots normalized to actin. (Unpaired t-test, two-tailed; n=4 mice per group)

b SynCAM 1 increase in SPM from frontal cortex upon LRRTM1 loss compared to WT. Left, region for fractionation in **b, c** marked in red. Center, immunoblots of SPMs from the frontal cortex of WT and LRRTM1 KO littermate mice. Dashed lines mark bands from the same blot exposure. Right, SynCAM 1 quantification normalized to GAPDH. (Unpaired t-test, two-tailed; n=4 WT, 3 LRRTM1 KO fractions, each one male mouse)

c Increased abundance of LRRTM1 in frontal cortex SPM prepared from SynCAM 1 KO compared to WT. Left, immunoblots of SPMs from the frontal cortex of WT and SynCAM 1 KO littermate mice. Dashed lines separate lanes from the same blot exposure. Right, quantification of LRRTM1 normalized to GAPDH. (Unpaired t-test, two-tailed; t-test; n=4 WT, 3 LRRTM1 KO fractions, each one male mouse)

* $p<0.05$. Error bars show Standard Error of the Mean. M_r provided in kilodaltons.

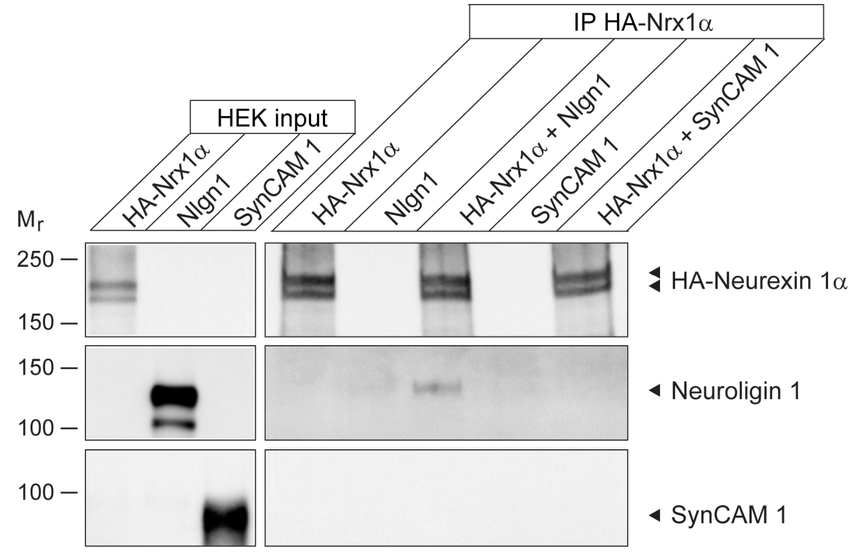

Supplementary Figure 2. Analysis of Neurexin interactions.

HEK293 cells were individually transfected with full-length constructs for HA-tagged Neurexin 1α, Neuroligin 1, or SynCAM 1. Left panel, 2.5 µg of total input detergent extracts from each transfection condition were loaded. Right panel, immunoprecipitates obtained with antibodies against the HA epitope from extracts of cells expressing individual proteins or after combining extracts as indicated. Loaded eluates correspond to 50 µg of individual conditions, equivalent to 5% input. HA and SynCAM 1 panels are from the same immunoblots at the same exposures, and Neuroligin 1 panels are cropped from the same immunoblot scanned at different exposures; see source data appendix for reference. Neuroligin 1 was co-immunoprecipitated with HA-tagged Neurexin 1α as expected. No interaction with SynCAM 1 was observed. M_r provided in kilodaltons.

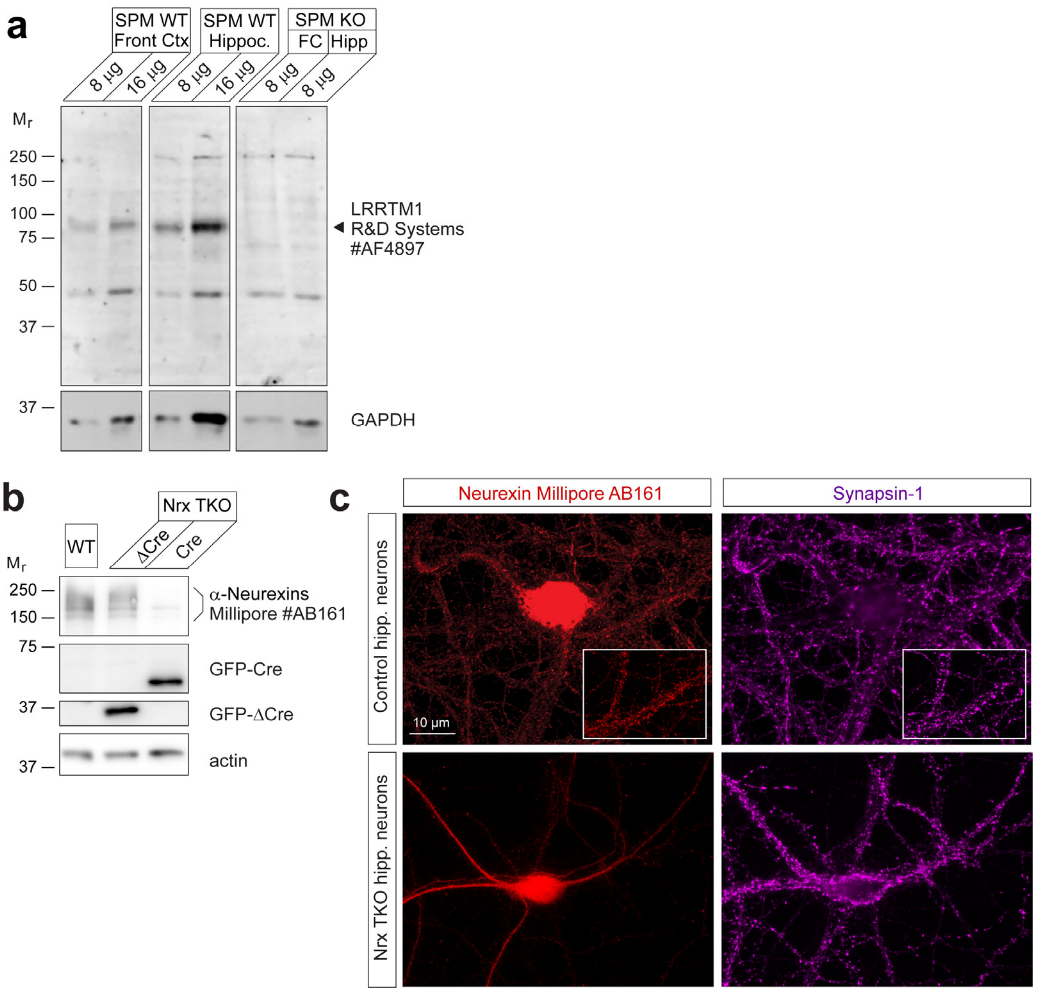

Supplementary Figure 3. Specificity of LRRTM1 and Neurexin antibodies.

a Specificity of LRRTM1 antibodies used in this study in immunoblotting. Synaptic plasma membranes (SPM) were purified from the frontal cortex (FC) or hippocampus of WT mice (lanes 1-4) and LRRTM1 KO littermates (lanes 5, 6). Protein amounts of 8 µg or 16 µg were loaded, and blot membranes were probed for LRRTM1. GAPDH served as loading control. All lanes shown were from the same blot at the same exposure. Blots are representative of results obtained in independent experiments. M_r provided in kilodaltons.

b Specificity of pan-Neurexin antibody Millipore #AB161 in immunoblotting. Total cell lysates were obtained from dissociated cultures of WT or Neurexin 1/2/3 $^{fl/fl}$ conditional triple KO (cTKO) hippocampal neurons transduced with lentivirus delivering GFP-Cre or inactive GFP-ΔCre as control. Samples were prepared at 21 div. Blots were probed with the indicated antibodies. GFP-ΔCre migrates at a lower molecular weight compared to GFP-Cre as part of the recombinase is deleted for inactivation. Actin served as loading control. Blots are representative of results obtained in three independent experiments.

c Specificity of pan-Neurexin antibody Millipore #AB161 in immunostaining. Panels show Neurexin 1/2/3 $^{fl/fl}$ cTKO hippocampal neurons that had been transduced with lentivirus delivering an inactive GFP-ΔCre (control, top row) or GFP-Cre (TKO, bottom row). Neurons were immunostained for Neurexins (red) and presynaptic Synapsin-1 (magenta) at 21 div. Confocal images are shown, and insets depict enlarged areas. The punctate Neurexin labeling along neurites in control neurons was not observed after Cre delivery and specific. Non-specific, diffuse staining remained in cell bodies and neurites after Cre deletion. Images are representative of results in four independent experiments. Neurexin 1/2/3 cTKO mice could not be analyzed by immunohistochemistry due to postnatal lethality. Scale bar in overview, 10 µm.

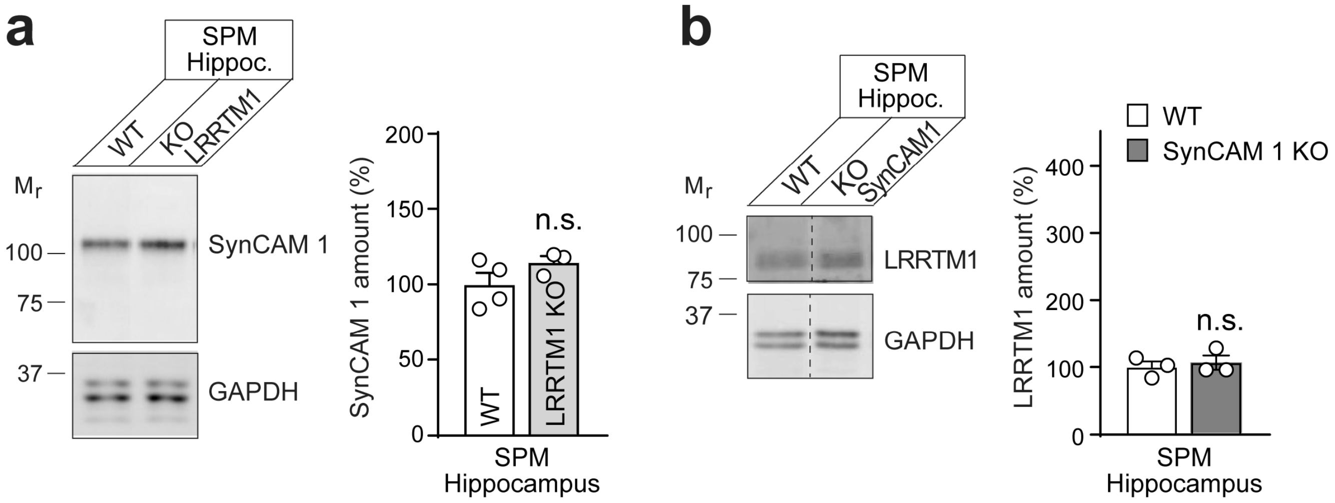

Supplementary Figure 4. Unchanged expression of SynCAM 1 and LRRTM1 in hippocampal synaptic plasma membranes from LRRTM1 and SynCAM 1 KO mice.

a No change in SynCAM 1 amount in synaptic plasma membranes (SPM) purified from LRRTM1 KO hippocampus compared to WT. Left, immunoblots of SPM from WT and LRRTM1 KO probed for SynCAM 1 and GAPDH as loading control. Right, SynCAM 1 quantification normalized to GAPDH. (Unpaired t-test, two-tailed; n=4 WT, 3 LRRTM1 KO mice)

b Unchanged LRRTM1 abundance in SPMs from SynCAM 1 KO hippocampus compared to WT. Left, SPM immunoblots for the indicated proteins. Dashed lines separate lanes from the same blot exposure. Right, LRRTM1 quantification normalized to loading control. (Unpaired t-test, two-tailed; n=3 WT, 3 LRRTM1 KO mice)

n.s., not significant. Error bars show Standard Error of the Mean. M_r provided in kilodaltons.

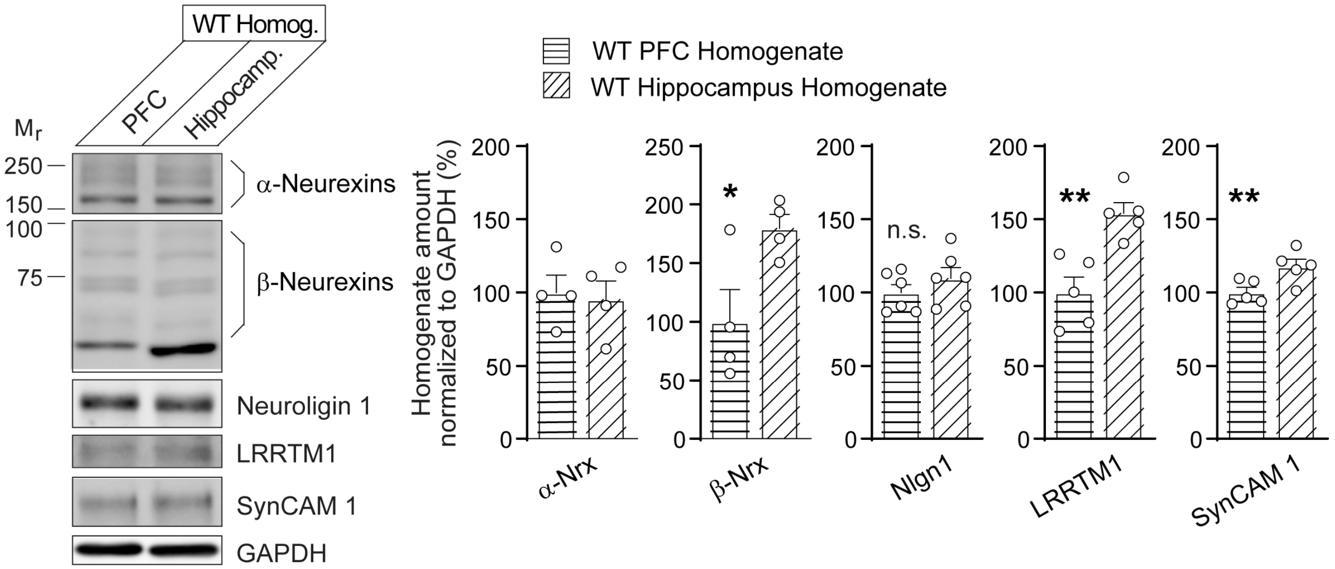

Supplementary Figure 5. Expression of synapse-organizing adhesion molecules in WT PFC and hippocampus. Neurexin 1β, LRRTM1, and SynCAM 1 are less abundant in homogenates prepared from WT PFC than WT hippocampus. Left, immunoblots of homogenates probed for the indicated proteins. 20 μg protein was loaded per lane. Right, quantification of immunoblots probed for the indicated proteins normalized to GAPDH. (Unpaired t-test, two-tailed; n=4-5 mice per region)

* $p<0.05$, ** $p<0.01$. Error bars show Standard Error of the Mean. M_r provided in kilodaltons.

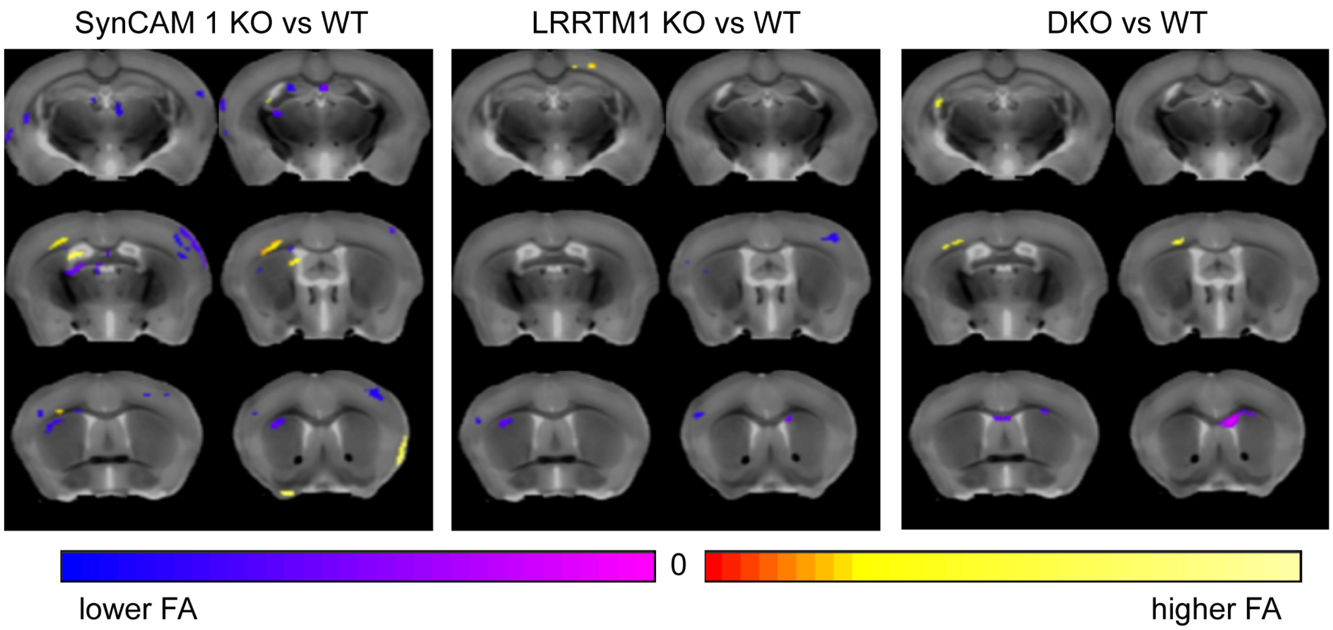

Supplementary Figure 6. MRI analysis of brain anatomy.

Single and double KO brains exhibit no gross anatomical and connectivity differences. Maps of voxel-based analysis of mean fractional anisotropy (FA) are shown. Diffusion tensor imaging (DTI) tracks major fiber tracts by quantifying the directional diffusion of water molecules as FA (Chahboune, H. et al. *NMR Biomed* 20, 375-382 (2007)). Datasets were obtained from anesthetized mice and registered to a reference anatomical image. Trends towards decreased and increased FA of single KO and DKO mice compared to WT mice are shown in blue and yellow, respectively. Brain-wide exploratory analysis observed trends towards global FA decreases and FA decreases in cerebral cortex in SynCAM 1 KO and LRRTM1 KO mice compared to WT and increased FA in cerebral cortex of DKO mice compared to WT, but no significant group by hemisphere interaction and no significant main effect of group was determined after correction for multiple comparisons. (n=5 WT, 5 SynCAM 1 KO, 6 LRRTM1 KO, 5 DKO)

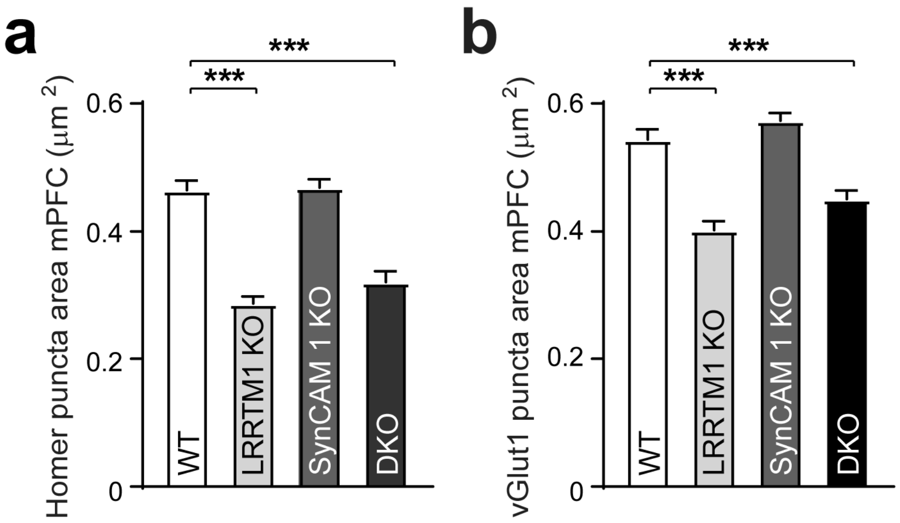

Supplementary Figure 7. Loss of LRRTM1 reduces the size of excitatory synaptic specializations in the mPFC.

Quantification of images as in Figure 2d shows that the area of excitatory postsynaptic Homer puncta (a) and presynaptic vGlut1 puncta (b) is reduced in LRRTM1 KO mice. No change was observed upon loss of SynCAM 1. (One-way ANOVA with post-hoc Tukey's test; n=35 ROI from 4 WT, 35 from 4 SynCAM 1 KO, 64 from 4 LRRTM1 KO, and 64 from 4 DKO)

*** $p<0.001$. Error bars show Standard Error of the Mean.

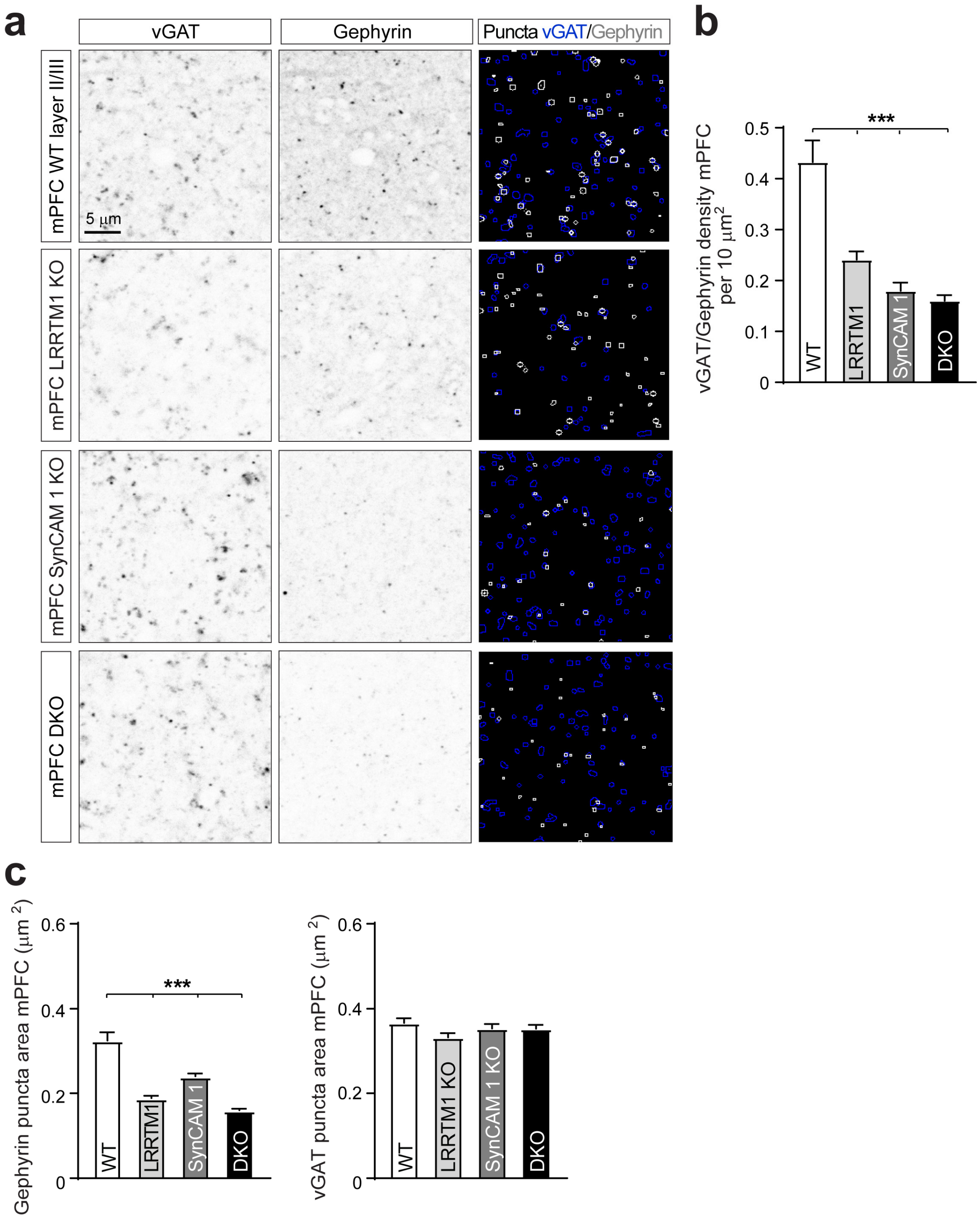

Supplementary Figure 8. Inhibitory synapse changes in the mPFC upon loss of LRRTM1 and SynCAM 1.

a Immunostainings of mPFC layer II/III for inhibitory presynaptic vGAT and inhibitory postsynaptic Gephyrin. Right column, algorithm-traced vGAT and Gephyrin puncta (blue and white, respectively, in the merge).

b The density of inhibitory synapses, defined as sites where vGAT and Gephyrin puncta co-localize, is reduced in both single and double KO mice. Images as in **a** were quantified. (One-way ANOVA with post-hoc Tukey's test; n=40 ROI WT, 38 SynCAM 1 KO, 39 LRRTM1 KO, and 39 DKO)

c Quantification of images as in **a** shows that the area of inhibitory postsynaptic Gephyrin puncta (left) is reduced in single and double LRRTM1 and SynCAM 1 KO mice. No change in the area of presynaptic vGAT puncta was observed (right). Statistical test and number of analyzed ROI as in **b**.

*** $p<0.001$. Error bars show Standard Error of the Mean.

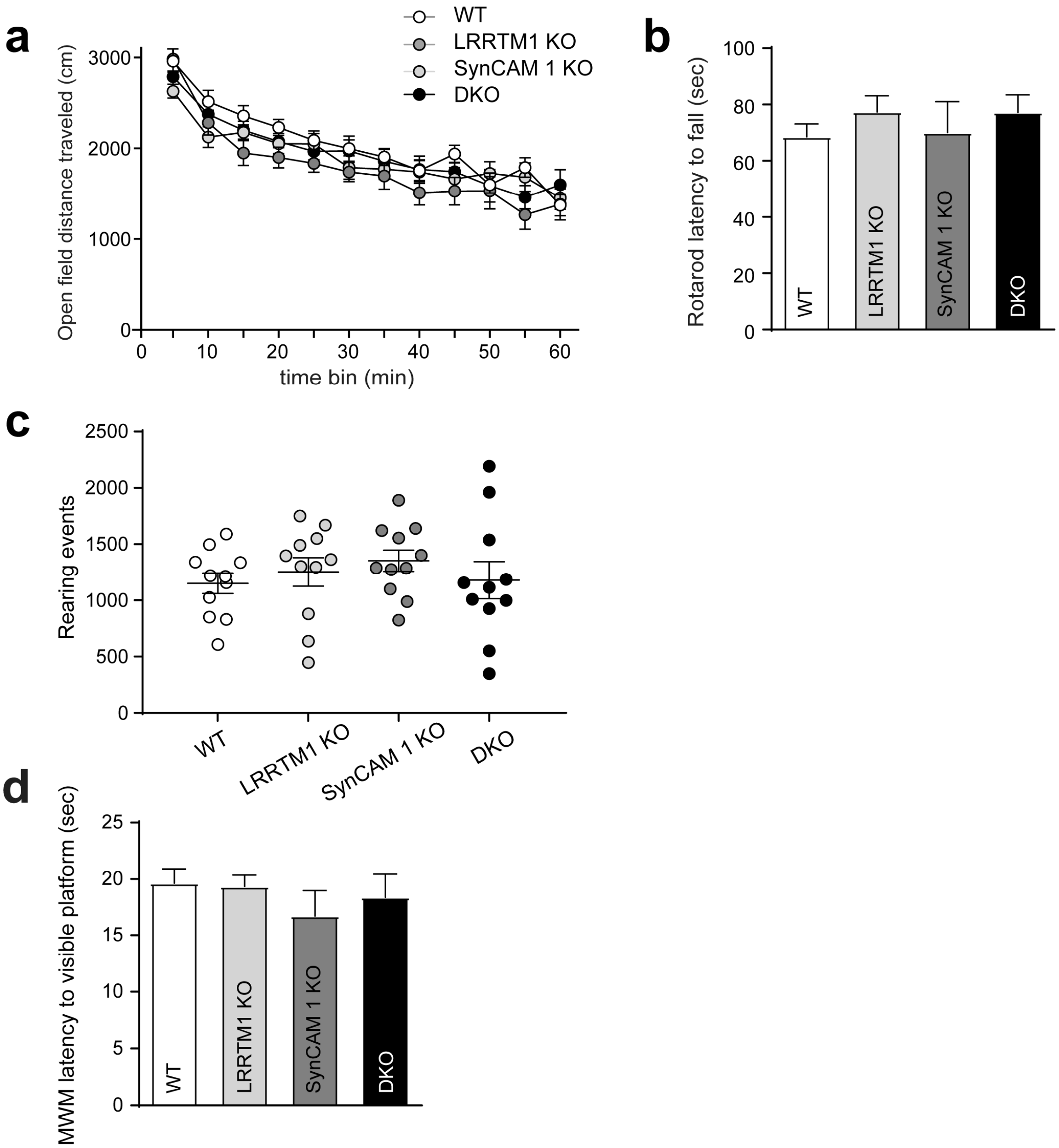

Supplementary Figure 9. Behavioral results and controls.

a Locomotor activity, as measured by distance traveled in an open field over 60 min and habituation of motor activity are not altered by single or combined loss of LRRTM1 and SynCAM 1 compared to WT. (n=11 WT, 11 LRRTM1 KO, 11 SynCAM 1 KO, and 11 DKO mice)

b Motor coordination and learning are not affected in LRRTM1 and SynCAM 1 single or double KO mice compared to WT as determined by measuring the cumulative time for which each mouse maintains balance on the accelerating Rotarod prior to falling off. (n=18 WT, 10 LRRTM1 KO, 8 SynCAM 1 KO, and 9 DKO mice)

c Rearing behavior in the open field is not altered in LRRTM1 and SynCAM 1 single or double KO mice compared to WT. (n=11 WT, 11 LRRTM1 KO, 11 SynCAM 1 KO, and 11 DKO mice)

d Mice tested in the Morris water maze were equally able to locate and reach the escape platform visually marked with a flag. (n=14 WT, 14 LRRTM1 KO, 8 SynCAM 1 KO, and 10 DKO mice)

Error bars show the Standard Error of the Mean.
