# Supplementary material for: Concerted roles of LRRTM1 and SynCAM 1 in organizing prefrontal cortex synapses and cognitive functions
Source: Nat Commun. 2023 Jan 28;14:459. doi: 10.1038/s41467-023-36042-w (PMC9884278; doi:10.1038/s41467-023-36042-w)
Supplement: Supplementary file 3 — Reporting Summary [file 41467_2023_36042_MOESM3_ESM.pdf]

nature portfolio

Corresponding author(s): Thomas Biederer

Last updated by author(s): Jan 6, 2023

Reporting Summary

Nature Portfolio wishes to improve the reproducibility of the work that we publish. This form provides structure for consistency and transparency in reporting. For further information on Nature Portfolio policies, see our Editorial Policies and the Editorial Policy Checklist.

Statistics

For all statistical analyses, confirm that the following items are present in the figure legend, table legend, main text, or Methods section.

n/a	Confirmed	
☐	☒	The exact sample size (*n*) for each experimental group/condition, given as a discrete number and unit of measurement
☒	☐	A statement on whether measurements were taken from distinct samples or whether the same sample was measured repeatedly
☐	☒	The statistical test(s) used AND whether they are one- or two-sided<br>*Only common tests should be described solely by name; describe more complex techniques in the Methods section.*
☒	☐	A description of all covariates tested
☐	☒	A description of any assumptions or corrections, such as tests of normality and adjustment for multiple comparisons
☐	☒	A full description of the statistical parameters including central tendency (e.g. means) or other basic estimates (e.g. regression coefficient) AND variation (e.g. standard deviation) or associated estimates of uncertainty (e.g. confidence intervals)
☐	☒	For null hypothesis testing, the test statistic (e.g. *F*, *t*, *r*) with confidence intervals, effect sizes, degrees of freedom and *P* value noted<br>*Give P values as exact values whenever suitable.*
☒	☐	For Bayesian analysis, information on the choice of priors and Markov chain Monte Carlo settings
☒	☐	For hierarchical and complex designs, identification of the appropriate level for tests and full reporting of outcomes
☐	☒	Estimates of effect sizes (e.g. Cohen's *d*, Pearson's *r*), indicating how they were calculated

Our web collection on statistics for biologists contains articles on many of the points above.

Software and code

Policy information about availability of computer code

Data collection	• Microscopy: Leica Application Suite X<br>• Biochemistry immunoblots: ProteinSimple AlphaView<br>• Behavioral studies: Kinder Scientific MotorMonitor, Kinder Scientific StartleMonitor, Noldus Ethovision 15<br>• MRI: B0DETOX, Varian VNMRJ 2.2b<br>• Electrophysiology: Cambridge Electronic Design Spike2
Data analysis	• Microscopy: Leica Zen, custom MATLAB code, ImageJ Version 1.53v<br>• Biochemistry: ImageJ<br>• MRI: SPM8, BioImage Suite 3.01, custom MATLAB code<br>• Statistical analyses: GraphPad Prism 9.4.1

For manuscripts utilizing custom algorithms or software that are central to the research but not yet described in published literature, software must be made available to editors and reviewers. We strongly encourage code deposition in a community repository (e.g. GitHub). See the Nature Portfolio guidelines for submitting code & software for further information.

Data

Policy information about availability of data

All manuscripts must include a data availability statement. This statement should provide the following information, where applicable:

- Accession codes, unique identifiers, or web links for publicly available datasets
- A description of any restrictions on data availability
- For clinical datasets or third party data, please ensure that the statement adheres to our policy

A source data file for the figures is provided with this paper, including unprocessed scans of immunoblots and all data points shown in graphs. Because of their size, the raw microscopy images, the electrophysiological recordings, and the MRO scans obtained and analyzed in this study will be made available upon request to the corresponding author. The Supplemental Data includes the source data for the supplementary immunoblots.

Human research participants

Policy information about studies involving human research participants and Sex and Gender in Research.

Reporting on sex and gender	*Use the terms sex (biological attribute) and gender (shaped by social and cultural circumstances) carefully in order to avoid confusing both terms. Indicate if findings apply to only one sex or gender; describe whether sex and gender were considered in study design whether sex and/or gender was determined based on self-reporting or assigned and methods used. Provide in the source data disaggregated sex and gender data where this information has been collected, and consent has been obtained for sharing of individual-level data; provide overall numbers in this Reporting Summary. Please state if this information has not been collected. Report sex- and gender-based analyses where performed, justify reasons for lack of sex- and gender-based analysis.*
Population characteristics	*Describe the covariate-relevant population characteristics of the human research participants (e.g. age, genotypic information, past and current diagnosis and treatment categories). If you filled out the behavioural & social sciences study design questions and have nothing to add here, write "See above."*
Recruitment	*Describe how participants were recruited. Outline any potential self-selection bias or other biases that may be present and how these are likely to impact results.*
Ethics oversight	*Identify the organization(s) that approved the study protocol.*

Note that full information on the approval of the study protocol must also be provided in the manuscript.

Field-specific reporting

Please select the one below that is the best fit for your research. If you are not sure, read the appropriate sections before making your selection.

[x] Life sciences [ ] Behavioural & social sciences [ ] Ecological, evolutionary & environmental sciences

For a reference copy of the document with all sections, see nature.com/documents/nr-reporting-summary-flat.pdf

Life sciences study design

All studies must disclose on these points even when the disclosure is negative.

Sample size	For biochemical, imaging, and electrophysiological analyses, sample sizes are comparable with or larger than those previously reported in studies with similar experimental designs. For behavioral analyses, the sample size was approximately 10-18 mice for each genotype, comparable with or larger than those previously reported studies with similar experimental designs. Sample sizes are reported throughout the figure legends.
Data exclusions	No data were excluded.
Replication	At least three independent biological repeats were conducted for all biochemical, microscopy and electrophysiology experiments. Data representative for the results are shown in the figures.
Randomization	In behavioral and electrophysiological experiments, mice of experimental and control groups were randomly counterbalanced. Randomization was not relevant for other experiments as data were acquired in parallel for each group.
Blinding	Data collection and analysis were performed blind to genotype or experimental group as described in the Methods.

Reporting for specific materials, systems and methods

We require information from authors about some types of materials, experimental systems and methods used in many studies. Here, indicate whether each material, system or method listed is relevant to your study. If you are not sure if a list item applies to your research, read the appropriate section before selecting a response.

Materials & experimental systems

n/a	Involved in the study
☐	☒ Antibodies
☐	☒ Eukaryotic cell lines
☐	☐ Palaeontology and archaeology
☐	☒ Animals and other organisms
☐	☐ Clinical data
☐	☐ Dual use research of concern

Methods

n/a	Involved in the study
☐	☐ ChIP-seq
☐	☐ Flow cytometry
☐	☒ MRI-based neuroimaging

Antibodies

Antibodies used

The primary antibodies used in this study are listed below and this information is provided in the Methods section, together with the application-specific dilution information:
• Immunoblotting: LRRTM1 (R&D Systems Cat# AF4897, RRID:AB_10643427), SynCAM 1 (MBL Laboratories Cat# CM004-3, clone 3E1, RRID:AB_592783), Neurexins (Millipore Cat# ABN161, RRID:AB_10917110), Neuroligin 1 (Synaptic Systems Cat#129 111, clone 4C12, RRID:AB_887747), Neuroligin 3 (Neuromab clone N110/29, RRID:MMRRC_066080-UCD), N-CAM 180 (Sigma Cat# C9672, RRID:AB_1079450), and GAPDH (Millipore Cat# MAB374, clone 6C5, AB_2107445). Secondary antibodies were conjugated with AlexaFluor dyes 488, 555, or 647 (Thermo Fisher).
• Immunohistochemistry: vGlut1 (NeuroMab clone N28/9, RRID:MMRRC_065995-UCD), Homer 1/2/3 (Synaptic Systems Cat# 160 003, RRID:MMRRC_065995-UCD), Neurexins (Millipore Cat# ABN161, RRID:AB_10917110), SynCAM 1 (MBL Laboratories Cat# CM004-3, clone 3E1, RRID:AB_592783), PSD-95 (Cell Signaling Cat# 3409, RRID:AB_1264242), Parvalbumin (Swant, Cat# PVG-213, RRID:AB_2650496; 1:500), and MAP2 (Millipore Cat# MAB3418, RRID:AB_94856). Secondary antibodies were conjugated with AlexaFluor dyes 488, 555, or 647 (Thermo Fisher).
• Immunocytochemistry: Synapsin 1 (Synaptic Systems Cat# 106 001, RRID:AB_887805), Neurexin (Millipore Cat# ABN161, RRID:AB_10917110), and CyTM3-conjugated goat-anti-rabbit IgG (Jackson ImmunoResearch Cat# 111-165-003, RRID:AB_2338000)

Validation

Antibodies against LRRTM1 and Neurexin were validated in this study using mouse knock-out samples for immunoblotting and immunostaining applications as shown in Supplemental Figure 2. Antibodies against SynCAM 1 were previously validated for immunoblotting and immunohistochemical staining applications using mouse knock-out brain tissue samples in Giza, J.I., et al. Neuropsychopharmacology 38, 628-638 (2013) and Ribic, A., Crair, M.C. & Biederer, T. Cell Rep 26, 381-393 e386 (2019). Validation of other antibodies used in this study is provided on the manufacturer websites and references in the SYNGO database https://www.syngoportal.org/

Eukaryotic cell lines

Policy information about cell lines and Sex and Gender in Research

Cell line source(s)	HEK293 (ATCC Cat# CRL-1573, RRRID:CVCL_0045)
Authentication	Cells were directly purchased from ATCC.
Mycoplasma contamination	No mycoplasma contamination was detected in our routine checks.
Commonly misidentified lines (See ICLAC register)	*Name any commonly misidentified cell lines used in the study and provide a rationale for their use.*

Palaeontology and Archaeology

Specimen provenance	*Provide provenance information for specimens and describe permits that were obtained for the work (including the name of the issuing authority, the date of issue, and any identifying information). Permits should encompass collection and, where applicable, export.*
Specimen deposition	*Indicate where the specimens have been deposited to permit free access by other researchers.*
Dating methods	*If new dates are provided, describe how they were obtained (e.g. collection, storage, sample pretreatment and measurement), where they were obtained (i.e. lab name), the calibration program and the protocol for quality assurance OR state that no new dates are provided.*

☐ Tick this box to confirm that the raw and calibrated dates are available in the paper or in Supplementary Information.

Ethics oversight	*Identify the organization(s) that approved or provided guidance on the study protocol, OR state that no ethical approval or guidance was required and explain why not.*

Note that full information on the approval of the study protocol must also be provided in the manuscript.

Animals and other research organisms

Policy information about studies involving animals; ARRIVE guidelines recommended for reporting animal research, and Sex and Gender in Research

Laboratory animals	8-10 week old mice of both sexes on a C57BL/6NCrl background were used for all experiments. Mice were housed in an environment of between 40%-60% relative humidity and an ambient temperature of approximately 21 °C/70 °F. Constitutive SynCAM 1 KO mice, constitutive LRRTM1 KO, and conditional Neurexin 1/2/3 fl/fl KO mice were analyzed.
Wild animals	This study did not involve wild animals.
Reporting on sex	Animals of both sexes were analyzed except for behavioral experiments, where only male mice were tested. Data were not disaggregated by sex.
Field-collected samples	This study did not involve field collected samples.
Ethics oversight	All animal procedures undertaken in this study were approved by Institutional Animal Care and Use Committees (Tufts University, Boston, Massachusetts, USA and Yale University, New Haven, Connecticut, USA) in compliance with NIH guidelines and the Landesamt für Natur, Umwelt und Verbraucherschutz (North Rhine-Westphalia, Germany).

Note that full information on the approval of the study protocol must also be provided in the manuscript.

Clinical data

Policy information about clinical studies

All manuscripts should comply with the ICMJEguidelines for publication of clinical research and a completedCONSORT checklist must be included with all submissions.

Clinical trial registration	*Provide the trial registration number from ClinicalTrials.gov or an equivalent agency.*
Study protocol	*Note where the full trial protocol can be accessed OR if not available, explain why.*
Data collection	*Describe the settings and locales of data collection, noting the time periods of recruitment and data collection.*
Outcomes	*Describe how you pre-defined primary and secondary outcome measures and how you assessed these measures.*

Dual use research of concern

Policy information about dual use research of concern

Hazards

Could the accidental, deliberate or reckless misuse of agents or technologies generated in the work, or the application of information presented in the manuscript, pose a threat to:

No	Yes	
☒	☐	Public health
☒	☐	National security
☒	☐	Crops and/or livestock
☒	☐	Ecosystems
☒	☐	Any other significant area

Experiments of concern

Does the work involve any of these experiments of concern:

No	Yes	
☒	☐	Demonstrate how to render a vaccine ineffective
☒	☐	Confer resistance to therapeutically useful antibiotics or antiviral agents
☒	☐	Enhance the virulence of a pathogen or render a nonpathogen virulent
☒	☐	Increase transmissibility of a pathogen
☒	☐	Alter the host range of a pathogen
☒	☐	Enable evasion of diagnostic/detection modalities
☒	☐	Enable the weaponization of a biological agent or toxin
☒	☐	Any other potentially harmful combination of experiments and agents

ChIP-seq

Data deposition

☐ Confirm that both raw and final processed data have been deposited in a public database such as GEO.

☐ Confirm that you have deposited or provided access to graph files (e.g. BED files) for the called peaks.

Data access links<br>*May remain private before publication.*	*For "Initial submission" or "Revised version" documents, provide reviewer access links. For your "Final submission" document, provide a link to the deposited data.*
Files in database submission	*Provide a list of all files available in the database submission.*
Genome browser session<br>(e.g. UCSC)	*Provide a link to an anonymized genome browser session for "Initial submission" and "Revised version" documents only, to enable peer review. Write "no longer applicable" for "Final submission" documents.*

Methodology

Replicates	*Describe the experimental replicates, specifying number, type and replicate agreement.*
Sequencing depth	*Describe the sequencing depth for each experiment, providing the total number of reads, uniquely mapped reads, length of reads and whether they were paired- or single-end.*
Antibodies	*Describe the antibodies used for the ChIP-seq experiments; as applicable, provide supplier name, catalog number, clone name, and lot number.*
Peak calling parameters	*Specify the command line program and parameters used for read mapping and peak calling, including the ChIP, control and index files used.*
Data quality	*Describe the methods used to ensure data quality in full detail, including how many peaks are at FDR 5% and above 5-fold enrichment.*
Software	*Describe the software used to collect and analyze the ChIP-seq data. For custom code that has been deposited into a community repository, provide accession details.*

Flow Cytometry

Plots

Confirm that:

☐ The axis labels state the marker and fluorochrome used (e.g. CD4-FITC).

☐ The axis scales are clearly visible. Include numbers along axes only for bottom left plot of group (a 'group' is an analysis of identical markers).

☐ All plots are contour plots with outliers or pseudocolor plots.

☐ A numerical value for number of cells or percentage (with statistics) is provided.

Methodology

Sample preparation	*Describe the sample preparation, detailing the biological source of the cells and any tissue processing steps used.*
Instrument	*Identify the instrument used for data collection, specifying make and model number.*
Software	*Describe the software used to collect and analyze the flow cytometry data. For custom code that has been deposited into a community repository, provide accession details.*
Cell population abundance	*Describe the abundance of the relevant cell populations within post-sort fractions, providing details on the purity of the samples and how it was determined.*
Gating strategy	*Describe the gating strategy used for all relevant experiments, specifying the preliminary FSC/SSC gates of the starting cell population, indicating where boundaries between "positive" and "negative" staining cell populations are defined.*

☐ Tick this box to confirm that a figure exemplifying the gating strategy is provided in the Supplementary Information.

Magnetic resonance imaging

Experimental design

Design type	Structural imaging, diffusion tensor imaging

Design specifications | Resting state fMRI 2048 s divided into 4 sessions of 512 s (no break between sessions)

Behavioral performance measures | N/A

Acquisition

Imaging type(s) | Resting state fMRI; structural imaging, diffusion tensor imaging

Field strength | 9.4T

Sequence & imaging parameters | Resting state fMRI: GE-EPI 25.6x12.8mm FOV, 64x32 matrix, 8x1 mm slices, TR 2000 ms, TE 13 ms.
Structural imaging: FSEMS 25.6x12.8mm FOV, 128x64 matrix, 8x1 mm slices, 4000 ms TR, 48 ms TE, 90 deg flip angle.
Diffusion tensor imaging: TR =2s, TE= 26ms, 16 directions, one with no gradients, the other 15 with b=1000 s/mm2; image resolution was 256×256, 24 slices of 0.5 mm thickness, 4 averages, FOV 20 mm x 10 mm.

Area of acquisition | Whole brain excluding olfactory bulb and cerebellum

Diffusion MRI [x] Used [ ] Not used

Parameters | 15 directions with b=1000 s/mm2

Preprocessing

Preprocessing software | SPM8, BioImage Suite 3.01, Custom MATLAB code. No normalization, no segmentation, brain outlines and S1BF regions drawn manually per-slice per-mouse

Normalization | N/A

Normalization template | N/A

Noise and artifact removal | Data were slice-timing corrected, motion corrected, spatially blurred (sigma = 4 voxels). Results were tested both with and without regression of motion parameters, there was no significant difference (see text).

Volume censoring | N/A

Statistical modeling & inference

Model type and settings | No SPM, only individual signals tested (see text).

Effect(s) tested | Variance of whole-brain signal for resting state (see text).

Specify type of analysis: [x] Whole brain [ ] ROI-based [ ] Both

Statistic type for inference (See Eklund et al. 2016) | N/A

Correction | Sequential Goodness of Fit (binomial method, reference in text)

Models & analysis

n/a	Involved in the study
[ ]	[x] Functional and/or effective connectivity
[x]	[ ] Graph analysis
[x]	[ ] Multivariate modeling or predictive analysis

Functional and/or effective connectivity | Variance of whole-brain signal (see text) based on prior human schizophrenia studies (references in text).
